# TIMP-2 modulates cancer cell transcriptional profile and enhances E-cadherin/beta-catenin complex expression in A549 lung cancer cells

**DOI:** 10.18632/oncotarget.801

**Published:** 2013-01-27

**Authors:** Dimitra Bourboulia, HuiYing Han, Sandra Jensen-Taubman, Noah Gavil, Biju Isaac, Beiyang Wei, Len Neckers, William G. Stetler-Stevenson

**Affiliations:** ^1^ Radiation Oncology Branch, Center for Cancer Research, National Cancer Institute, Advanced Technology Center, 8717 Grovemont Circle, Bethesda, MD, USA; ^2^ Bowdoin College, Brunswick, ME, USA; ^3^ Center for Computational Science, University of Miami, Miami, FL, USA; ^4^ Urologic Oncology Branch, Center for Cancer Research, National Cancer Institute, Bethesda, MD, USA

**Keywords:** TIMP-2, microarray analysis, E-cadherin, cell adhesion, tumor growth inhibition

## Abstract

Tissue Inhibitor of Metalloproteinase 2 (TIMP-2) plays an essential role in regulating matrix remodeling, cell growth, differentiation, angiogenesis and apoptosis *in vitro* and *in vivo*. We have recently shown that TIMP-2-mediated inhibition of tumor growth is independent of matrix metalloproteinase-mediated mechanisms, and is a consequence of modulating both the tumor cells and the tumor microenvironment. In the current study we aim to identify the molecular pathways associated with these effects. We analyzed the transcriptional profile of the human lung cancer cell line A549 upon overexpression of TIMP-2 and Ala+TIMP-2 (mutant that does not inhibit MMP activity), and we found changes in gene expression predominantly related to decreased tumor development and metastasis. Increased E-cadherin expression in response to both TIMP-2 and Ala+TIMP-2 expression was confirmed by real time quantitative RT-PCR and immunoblotting. A549 cells treated with epidermal growth factor (EGF) displayed loss of cobblestone morphology and cell-cell contact, while cells overexpressing TIMP-2 or Ala+TIMP-2 were resistant to EGF-induced morphological changes. Moreover, exogenous treatment with recombinant Ala+TIMP-2 blocked EGF induced down-regulation of E-cadherin. *In vivo*, immunohistochemistry of A549 xenografts expressing either TIMP-2 or Ala+TIMP-2 demonstrated increased E-cadherin protein levels. More importantly, transcriptional profile analysis of tumor tissue revealed critical pathways associated with effects on tumor-host interaction and inhibition of tumor growth. In conclusion, we show that TIMP-2 promotes an anti-tumoral transcriptional profile *in vitro* and *in vivo*, including upregulation of E-cadherin, in A549 lung cancer cells.

## INTRODUCTION

TIMPs are a family of proteins that naturally inhibit the activity of matrix metalloproteinases (MMPs). Uncontrolled MMP proteolysis promotes extracellular matrix degradation, tumor cell invasion and increased risk for cancer cell metastasis [1-3]. TIMP-2 is a unique TIMP family member, in that it both inhibits active MMP-2 as well as activates pro-MMP-2 in the presence of a membrane tethered MMP, MMP14/MT1-MMP [4, 5]. TIMP-2 functions as an endogenous inhibitor of both angiogenesis and tumor growth, effects that may be dependent or independent of MMP inhibition [6-12]. Known mechanisms associated with inhibition of endothelial cell proliferation and migration *in vitro* and angiogenesis *in vivo* include: a) TIMP-2 binding to integrin α_3_β_1_ receptor and activation of SH2-containing protein tyrosine phosphatase-1 (SHP-1), which suppresses the activity of receptor tyrosine kinases VEGFR2 and FGFR1 upon growth factor stimulation (VEGF-A or FGF2); and b) C-terminus TIMP-2 loop 6 binding to insulin-like growth factor receptor I (IGF-IR) to disrupt downstream mitogenic signaling through AKT hypo-phosphorylation [9, 11, 13]. Major changes within downstream signal transduction pathways involve induction of cell cycle arrest with de novo synthesis of tumor suppressor gene p27^kip1^, retinoblastoma protein (pRb) hypo-phoshorylation, and up-regulation of the MMP inhibitor, reversion-inducing-cystein-rich protein with Kazal motif (RECK), via modification of paxillin phosphorylation and Rap1 up-regulation [14-18].

More recently, forced expression of TIMP-2 in A549 human lung cancer cells was performed to address whether TIMP-2 overexpression directly influences tumor angiogenesis and/or tumor cell behavior. Indeed, tumor cell migration and invasion were inhibited *in vitro*, whereas *in vivo* tumor growth inhibition was achieved through TIMP-2 mediated interaction with the tumor microenvironment, angiogenesis inhibition and induction of tumor cell apoptosis [12]. The use of Ala+TIMP-2 in similar experiments shows that mechanistically, inhibition of MMP activity does not entirely explain all TIMP-2 functions [19].

Therefore, it is apparent that TIMP-2 plays a broader role both in endothelial cell physiology and in cancer development [20]. In an attempt to understand how TIMP-2 regulates angiogenesis and tumor growth inhibition, we performed human cDNA microarray analysis and compared the differential gene expression profiles of A549 tumor cells overexpressing TIMP-2 or Ala+TIMP-2 with that of stably transfected Empty Vector control cells (EV) *in vitro* and *in vivo*. We found that the TIMP-2 transcriptional signature in A549 cells is associated with modulation of genes that inhibit endothelial cell proliferation, angiogenesis, lung cancer development and metastasis. Specifically, we show that the tumor suppressor gene E-cadherin is up-regulated by TIMP-2 overexpression *in vitro* and *in vivo*, contributing to the maintenance of cell-cell adhesion that may also contribute to inhibition of tumor growth.

## RESULTS AND DISCUSSION

### Differentially expressed gene profiles in TIMP-2 and Ala+TIMP-2 transfected A549 cell lines

**Figure 1:**
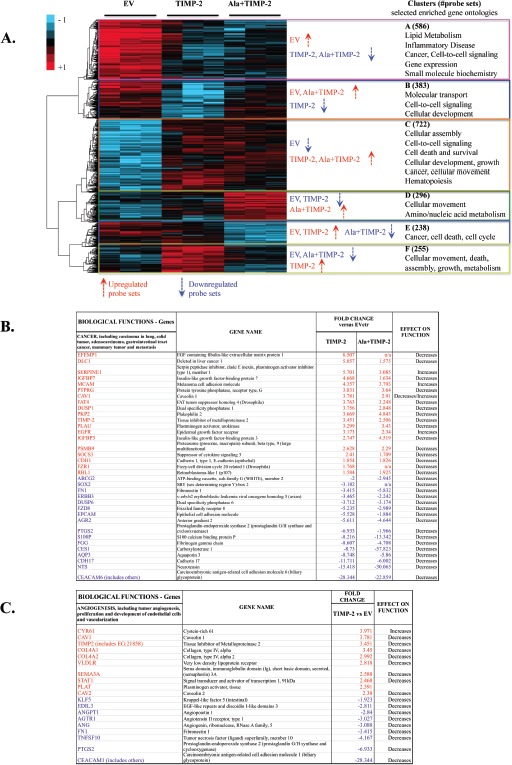
TIMP-2 is a transcriptional regulator of human lung cancer A549 cells *in vitro* (A) Clustered heat map diagram of microarray data for EV, TIMP-2 and Ala+TIMP-2 overexpressing A549 cells (three replicates). 2480 probe sets determined to significantly change across the three groups with false discovery rate (FDR) <10 are shown in the heat map. The key on the top left assigns heat map colors to the absolute gene expression value on a log_2_ scale. The most significant Gene Ontology (GO) categories, with the number of probe sets in parentheses, identifying enriched biological processes for each cluster across A549 TIMP-2 and Ala+TIMP-2 cells are shown next to the heat map. Bivariate comparisons of TIMP-2 (B and C) or Ala+TIMP-2 (B) with EV generated lists of functions unique for each comparison. Selected functions (cancer, B and angiogenesis, C) and transcriptional regulators with gene lists and predicted effect on function are shown. The most highly up-regulated (red) and down-regulated (blue) transcripts are presented with at least 1.5-fold change versus EV control.

To identify pathways resulting from TIMP-2 mediated effects on tumor cells and the microenvironment, we performed microarray analysis on TIMP-2 and Ala+TIMP-2 overexpressing A549 cells, previously shown to display reduced migration and invasion *in vitro*, and tumor growth and angiogenesis *in vivo* [12, 21]. The microarray data can be found at the link: http://www.ncbi.nlm.nih.gov/geo/query/acc.cgi?token=nxmzxmkaqgisipu&acc=GSE38408. Analysis of variance (ANOVA) of the data (see methods) identifies a subset of 2480 differentially expressed (DE) genes across the three A549 experimental groups: EV, TIMP-2 and Ala+TIMP-2 (Fig. 1A). Hierarchical clustering of DE genes using the ‘*Pearson centered*’ distance similarity method and ‘*average*’ linkage rule suggests that specific clusters of enriched gene functions belong exclusively to TIMP-2 (clusters B & F) or Ala+TIMP-2 (clusters D & E) groups, whereas other clusters (A & C) were common to both groups (Fig 1A). This analysis reveals several findings: the TIMP-2/Ala+TIMP-2 commonly expressed genes (clusters A & C) identify an MMP-independent function of TIMP-2, whereas genes that change predominantly in TIMP-2 overexpressing cells (clusters B & F) may a result of MMP inhibition. Finally, genes affected solely in the Ala+TIMP-2 group (clusters D & E) may depend on MMP activation.

Common AC genes in TIMP-2 or Ala+TIMP-2 samples were primarily associated with cellular movement, death, growth, signaling and metabolism (Supplementary Fig. 1A). Comprehensive analysis revealed that the most up- and down-regulated genes, in either TIMP-2 or Ala+TIMP-2 samples, were related to decreased cancer development and metastasis (Fig. 1B). More specifically, the secreted protein *EFEMP1* (up-regulated) was recently shown to suppress malignant glioma growth [22], and *DLC-1* (up-regulated) is a well-known tumor suppressor gene that regulates cell proliferation and migration [23]. On the other hand, *CEACAM6* (down-regulated) is overexpressed in a number of epithelial malignancies, particularly in invasive pancreatic cancer [24], and *NTS* (down-regulated) is shown to promote cancer cell growth [25]. In addition, decreased gene function associated with lung cancer development was shown in both TIMP-2 and Ala+TIMP-2 samples: *IGFBP3* and *CDH1* were up-regulated, and *S100P* and *PTGS2* were down-regulated [26-30]. These data indicate that TIMP-2 overexpression transcriptionally regulates tumor cell development and growth, independent of MMP inhibition, although further work is needed to identify specific mechanisms. TIMP-2 also exhibited significant changes related to angiogenesis inhibition and endothelial cell proliferation, including up-regulation of *CAV1*, *COL1A* and *SEMA3A*, and down-regulation of *CEACAM1*, *PTGS2*, and *FN1* (Fig. 1C) [31-37]. Taken together, these data suggest that TIMP-2 overexpression in tumor cells could inhibit angiogenesis through paracrine effects on tumor endothelium, while decreasing tumor growth directly through regulation of the tumor cell transcriptome. In addition, since Ala+TIMP-2 overexpression showed similar findings, our data may partially explain the MMP-independent activity exhibited by TIMP-2 (illustrated in previous and recent literature [9, 10, 12, 14, 18, 38-41]).

### TIMP-2 enhances E-cadherin expression and inhibits EGF-induced EMT

**Figure 2:**
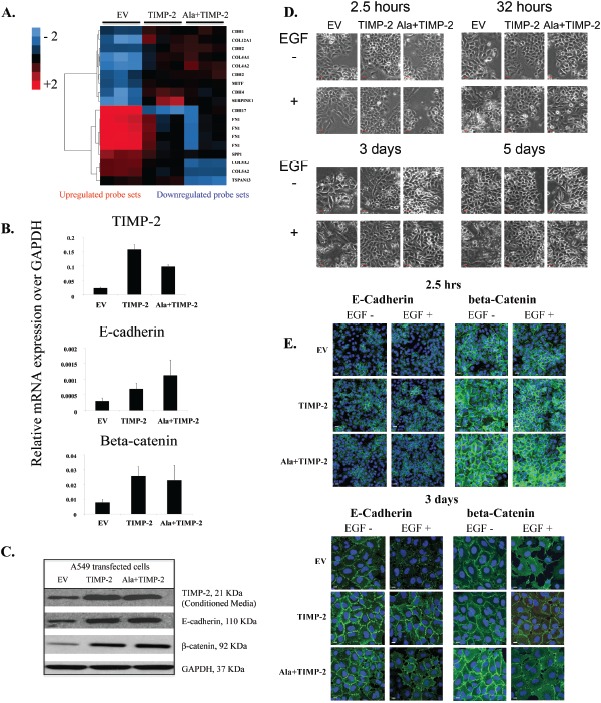
TIMP-2 overexpression upregulates E-cadherin and beta-catenin complex (A) A cDNA microarray analysis heat map showing differentially expressed genes related to EMT in A549 EV, TIMP-2 and Ala+TIMP-2 *in vitro* (FDR <5). The key on the top left assigns heat map colors to the absolute gene expression value on a log_2_ scale. (B) Real time quantitative RT-PCR and (C) Western blot were performed in A549 cells on selected genes and proteins. (D) Time course experiment showing morphological changes in A549 transfected cells untreated or treated with EGF for 2.5 hrs, 32 hrs, 3 days and 5 days (scale bars: 50 μm) and (E) immunofluorescence staining of E-cadherin and beta-catenin 2.5hrs and 3 days post EGF treatment.

One of the genes identified to be up-regulated in both TIMP-2 and Ala+TIMP-2 profiles is E-cadherin (*CDH1*), a gene expressed in most normal epithelial tissues. However, its loss in several carcinomas leads to increased tumor invasiveness, angiogenesis and epithelial to mesenchymal transition (EMT) [42, 43]. Therefore, we investigated the role of TIMP-2 in lung cancer development by analyzing genes associated with cell adhesion and EMT (Fig. 2A-C). Although some genes associated with mesenchymal phenotype also showed altered expression (*CDH2, SERPINE1*), E-cadherin was up-regulated in both TIMP-2 and Ala+TIMP-2 overexpressing A549 cells [44]. Since the C-terminus of E-cadherin binds directly to beta-catenin to stabilize cell-cell adhesion, we also examined beta-catenin expression (Fig. 2B, C) [45]. Indeed, both E-cadherin and beta-catenin were increased in TIMP-2 and Ala+TIMP-2 cells, both transcriptionally and at the protein level.

It has been previously demonstrated that treatment of A549 cells with EGF induces EMT via disruption of cell-cell adhesion, internalization of E-cadherin from cell-cell contacts, cellular morphological changes from cobblestone to spindle shape, and increased cell motility [46]. Here, treatment of A549 EV cells with EGF (100ng/ml) for 2.5 hours, 32 hours, 3 days and 5 days resulted in disruption of cell-cell adhesion and contacts (spindle shaped, elongated cells), whereas TIMP-2 and Ala+TIMP-2 overexpressing cells were resistant to chronic EGF treatment (3 and 5 days) (Fig. 2D). Additionally, previous studies demonstrated that reduced E-cadherin expression correlates with tumor invasion, metastasis and poor prognosis in patients with lung cancer [28, 47]. Here we show that both E-cadherin and beta-catenin are strongly localized at the plasma membrane and remain so after exposure to EGF in A549 TIMP-2 and Ala+TIMP-2, compared to EV cells (Fig. 2E). In contrast, EV cells treated with EGF showed reduced levels of both E-cadherin and beta-catenin expression at the plasma membrane and disrupted cell-cell adhesion. It was recently shown that TIMP-1 signaling promotes MDCK cell migration and invasion and induces an EMT program that also includes FAK activation [48]. TIMP-1, as opposed to TIMP-2, is overexpressed in a number of cancers, promoting tumor growth and inhibiting apoptosis, suggesting that the functional properties of TIMP family members are distinct. Indeed, we have previously shown that forced expression of TIMP-2 or Ala+TIMP-2 in A549 cells decreases both AKT and FAK phosphorylation in tumor xenografts [12].

### Exogenous treatment with Ala+TIMP-2 up-regulates E-cadherin in A549 cells and a linear correlation exists between TIMP-2 and E-cadherin mRNA levels in NSCLC cell lines

**Figure 3:**
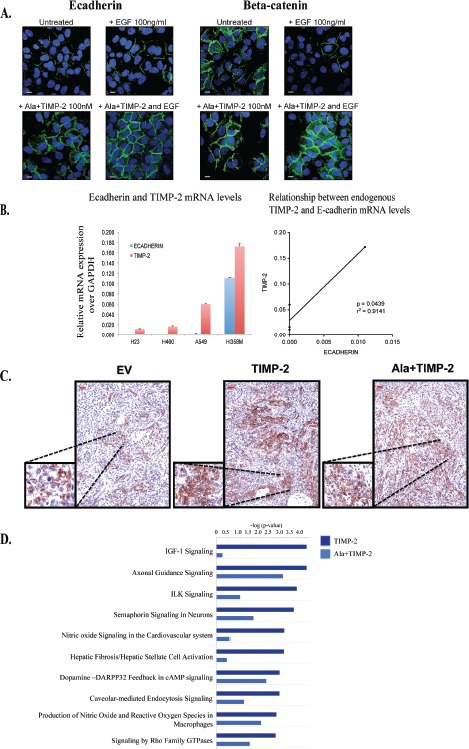
Exogenous treatment with Ala+TIMP-2, TIMP-2 expression in NCSLC and transcriptional analysis of tumor xenografts (A) Exogenous treatment with 100nM Ala+TIMP-2 on 24 hr serum starved A549 cells with and without additional treatment with 100ng/ml EGF for 2 hrs. Cells were stained with either E-cadherin (left) or beta-catenin (right) (scale bars: 50 μm). (B) Endogenous TIMP-2 gene expression levels have linear correlation with endogenous E-cadherin expression levels in 4 non-small cell lung cancer cell lines (NSCLC). (LEFT) The data presented as means+SEM of three independent experiments. Real time qRT-PCR analysis for *TIMP-2* and *E-cadherin* in 4 NSCLC (C) Expession of E-cadherin was determined by immunohistochemistry in A549 xenografts (scale bars: 50 μm, inserts 200 μm). Each photograph represents screening of 20 high power fields (at 100x magnification). (D) Comparisons between A549 TIMP-2 or Ala+TIMP-2 with EV were performed and a list of differentially expressed genes from each was taken for further study on Ingenuity Pathway Analysis (IPA) to identify enriched pathways. Top 10 canonical pathways activated by TIMP-2 and/or Ala+TIMP-2 are shown with a threshold value of <0.05 (yellow line).

Since stable transfection may alter cellular functionality, we addressed whether exogenous treatment of wild type A549 cells with Ala+TIMP-2 prior to addition of EGF would affect expression and localization of E-cadherin and beta-catenin (Fig. 3A). EGF treatment for 2 hrs decreased the levels of both beta-catenin and E-cadherin from the plasma membrane. However, Ala+TIMP-2 pretreatment for 10 min induced up-regulation of both E-cadherin and beta-catenin and, more importantly, prevented EGF induced down-regulation of E-cadherin and beta-catenin from the plasma membrane.

We then examined whether our findings are specific for A549 cells or whether other lung cancer cell lines show a similar pattern. We selected four non-small cell lung cancer (NSCLC) cell lines, H23, H460, A549 and H358M, and we determined the endogenous levels of both E-cadherin and TIMP-2 by real time PCR (Fig. 3B). Our results indicate that there is a linear co-relation between TIMP-2 and E-cadherin mRNA levels in these cells (p=0.0439, r^2^ = 0.9141), indicating that the expression of both molecules may be modulated by similar mechanisms.

### A549 xenografts show increased E-cadherin protein expression and display a transcriptional profile related to decreased tumor cell growth and metastasis

TIMP-2 and Ala+TIMP-2 A549 xenografts exhibited reduced tumor growth (70-95% inhibition), decreased angiogenesis and increased apoptosis in both nude and NOD-SCID mice (for tumor volumes and growth see [12, 21]). Immunohistochemical analysis of tumor tissue revealed increased E-cadherin expression in TIMP-2 and Ala+TIMP-2 xenografts (Fig. 3C). Previous data show that membrane loss of E-cadherin leads to cell-cell separation and promotes the angiogenic switch in lung cancer [43]. Taken together, our recent findings of decreased microvascular density in TIMP-2 and Ala+TIMP-2 xenografts indicate that novel additional mechanisms may contribute to the anti-angiogenic effects of TIMP-2 *in vivo* [12].

To understand the TIMP-2 effects on the tumor microenvironment and how these have contributed to the inhibition of tumor growth *in vivo,* we analyzed the transcriptional profiles of A549 TIMP-2 and Ala+TIMP-2 xenografts at day 21 post inoculation in NOD-SCID mice (Supplementary Fig 1B) [21]. TIMP-2 xenografts revealed several specific functions associated with decreased tumor metastasis and lipid metabolism, while Ala+TIMP-2 xenografts showed profiles associated with decreased tumor growth. In an attempt to explain the tumor growth inhibition in the TIMP-2 and Ala+TIMP-2 xenografts, canonical signaling pathways were analyzed (Fig. 3D). The most significant pathways, as determined by Ingenuity Pathway Analysis (IPA), included insulin-like growth factor-1 (IGF-1) signaling, axonal guidance signaling, integrin linked kinase (ILK) signaling, and semaphorin signaling in neurons (Fig. 3D). As shown, the IGF-1 canonical pathway, a network that regulates cellular proliferation and apoptosis in several cancers and is implicated in increased lung cancer risk, was negatively affected by TIMP-2 overexpression (Fig. 3D) [49, 50]. More specifically, when compared to EV tumors, TIMP-2 or Ala+TIMP-2 caused down-regulation of IGF-1 receptor (IGF-1R) expression (4.8 fold) and an increase in IGF binding proteins (including *IGFBP-*5 (4.0 fold) and -7 (3.5 fold)), proteins that limit the bioavailability of IGF ligands [51]. Given that inhibition of IGF-1R signaling leads to increased chemosensitivity in A549 cells, manipulation of the IGF signaling pathway may provide a useful therapeutic target for cancer therapy [52-55]. TIMP-2 has also been shown to inhibit IGF-1R directly by binding to the receptor expressed on endothelial cells [10, 11]. We, therefore, conclude that TIMP-2 acts on tumor cells and the tumor microenvironment to inhibit tumor growth.

**Figure 4:**
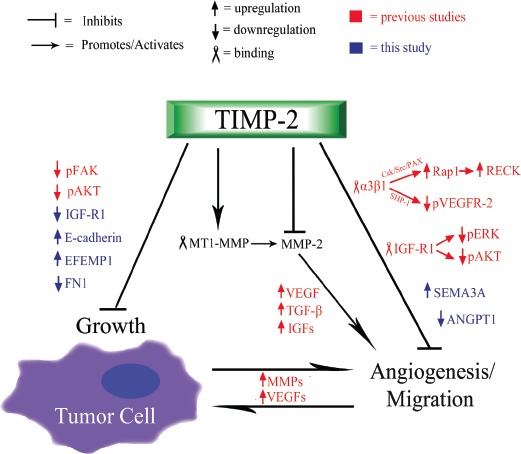
Schematic summary of TIMP-2 anti-tumoral and anti-angiogenic functions TIMP-2 is primarily understood to both inhibit and activate (via MMP-14/MT1-MMP) MMP-2, which breaks down the ECM, releasing various growth factors (e.g. VEGFs, TGF-β, IGFs) that promote angiogenesis and cell proliferation [1-5]. The process of angiogenesis further induces the up-regulation of MMPs and VEGFs, thus, creating a positive feedback that furthers vascular development and cell growth. Independent of MMP inhibition and activation, TIMP-2 binds to integrin α_3_β^−/−^ receptor, which causes signaling cascades (moderated by SHP-1 and Csk/Src/PAX/Rap1, respectively) that lead to the hypo-phosphorylation of VEGFR-2 and the up-regulation of the anti-migration factor, RECK [12, 20, 41]. Also, previous studies have shown that TIMP-2 binds to endothelial IGF-R1, which causes a net reduction in the phosphorylation of ERK and AKT, and the subsequent decrease in endothelial growth and angiogenesis [11]. Additional TIMP-2 induced, decreases in angiogenic function include the up-regulation of semaphorin-3A and the down-regulation of angiopoietin-1 (Fig 1C). Furthermore, TIMP-2 is associated with the inhibition of tumor, which has been paired with the observed hypo-phosphorylation of FAK and AKT, proteins that contribute to the proliferation and growth of tumor cells [12, 13]. Additionally, as presented in this study, TIMP-2 induces the up-regulation of E-cadherin, CDH1, which increases cell-cell adhesion, effectively inhibiting tumor cell growth, migration, and epithelial to mesenchymal transition (EMT). Finally, additional signs of TIMP-2 induced inhibition of tumor cell growth are the up-regulation of EFEMP1 and the down-regulation of fibronectin-1 (Fig 1B).

We, and others, have previously described MMP dependent and independent anti-angiogenic properties of TIMP-2 (Fig. 4). TIMP-2 can inhibit MMP-2 and overall migration and invasion of tumor endothelium. In addition, TIMP-2 inhibits blood vessel formation via receptor mediated mechanisms, including binding to integrin α_3_β_1_ on endothelial cells and inactivating receptor tyrosine kinases including VEGFR-2 and FGFR-2 through de-phosphorylation mediated by SHP-1. Additionally, TIMP-2 may also bind to IGFR-1 and decrease downstream signaling. In the current study, we have identified a novel anti-tumor activity of TIMP-2 – namely an ability to modify the transcriptional profile of tumor cells, resulting in increased E-cadherin expression and down-regulation of IGFR-1 signaling components. Decreased tumorigenesis and metastasis could be associated with increased E-cadherin expression in tumor cells, while inhibition of tumor angiogenesis may involve down-regulation of the IGFR-1 signaling pathway as well as tumor derived anti-angiogenic signals. Our study describes the anti-tumoral transcriptional signature of TIMP-2 in lung cancer cells.

## METHODS

### Cell culture and cell culture conditions

A549 cell culture, retroviral transfection and analysis were performed and comprehensive characterization and confirmation of inability of Ala+TIMP-2 to inhibit MMP has been previously shown [12]. A549 EV, TIMP-2 and Ala+TIMP-2 were grown to 80% confluency prior to replacing with fresh medium containing 0.5% fetal calf serum for 24 hours to induce quiescence. Cells were treated with or without EGF (100ng/ml) (BD Biosciences, San Jose, CA) for the indicated times (2.5 hours, 32 hours, 3 days and 5 days) and morphological changes were observed by phase-contrast microscopy (Olympus 1X70, Center Valley, PA). Exogenous treatment with Ala+TIMP-2 (100nM) was performed on 24-hours serum starved cells for 30min. EGF was added at 100ng/ml for 2 hours.

### Cell line, tumor RNA isolation and real time quantitative RT-PCR

Total RNA from three independent exponential growth phase cell cultures of A549 EV, TIMP-2, Ala+TIMP-2 and the 4 NSCLC cell lines was isolated using the RNAeasy kit (Qiagen, Gaithersburg, MD). Reverse transcription was performed as previously described [12]. Xenograft tumors were homogenized using QIAshredder (Invitrogen, Grand Island, NY) and RNA purified using the RNAeasy kit according to the manufacturer's instruction. Equal amounts of RNA (500ng) from six tumors per group were pooled into one sample and used to perform microarray analysis in tumor xenografts. *TIMP-2*, *GAPDH* primer sequences, real-time PCR conditions and analysis of gene expression were given elsewhere [12]. 500 nmol/L forward and reverse primers for *E-cadherin* and *beta-catenin* were used. Primer sequences for *E-cadherin*: forward, 5'- GGTCAGCGTGTGTGACTGTG -3' and reverse, 5'- GCAGAATCAGAATTAGCAAAGCAAG -3' and *Beta-catenin*: 5'- GCAGAAAATGGTTGCCTTGCT-3' and reverse, 5'- GCAGAAAATGGTTGCCTTGCT -3'. Mean CT values for target genes were normalized for the endogenous control *GAPDH*. The ratio of mRNA expression of target gene versus *GAPDH* was defined as 2(-ΔCt). All data are shown as mean ± S.E.M. of at least three independent experiments.

### Western blot analysis

Immunoblotting for TIMP-2 was performed in conditioned media (CM) as previously described [12]. Cell lysates were collected for analysis of E-cadherin and beta-catenin protein levels and probed overnight with mouse anti-E-cadherin antibody, 1/500 dilution (Abcam, Cambridge, MA) and mouse anti-beta-catenin antibody, 1/12,000 (BD Transduction, San Jose, CA).

### Immunofluorescence and Immunohistochemistry

For immunofluorescence studies, A549 cells were cultured on 4-well glass slides (NUNC, Rochester, NY), serum starved, and treated with EGF (100ng/ml) for 2.5 or 32 hours. Prior to staining, cells were fixed in 4% paraformaldehyde (Sigma) for 10 min, permeabilized in 0.1% Triton X-100 for 20 min, and blocked overnight in 1% BSA at 4°C with two washes in PBS at room temperature between each of the previous steps. Cells were incubated with mouse monoclonal antibodies to E-Cadherin (Abcam, 1:100) or Beta-catenin (Cell Signaling, 1:500) overnight at 4°C in a humidity chamber followed by incubation with goat anti mouse Alexa-488 secondary antibody (Cell Signaling, 1:800) for 1 hour at room temperature. Both primary and secondary antibodies were diluted in Antibody Diluent Solution (Dako, Carpenteria, CA). Representative images were captured using the Zeiss LSM510 Meta Confocal Microscope. The E-cadherin expression in resected tumors was determined by immunohistochemistry as previously described [12].

### Microarray analysis in A549 cell lines and xenograft tumors

Triplicate samples from A549 tumor cell lines representing 3 groups: EV ‘control’, TIMP-2 and Ala+TIMP-2 overexpressing cells were hybridized against the probes on Affymetrix HGU-133 Plus 2.0 chips. The array data were processed using the R/Bioconductor Statistical Package. MAS 5.0 normalization was performed on the probes and those with missing values were filtered. 20729 probes (sd ≥ 0.2) were retained for further analysis. Principal component analysis (PCA) and unbiased clustering on probe subsets were used to test the integrity of the arrays (data not shown). The ‘two-class unpaired’ t-test was performed using Significance of Microarray (SAM) module on R between samples TIMP-2 and EV, or Ala+TIMP-2 and EV individually to derive differentially expressed genes. ‘Multiclass’ ANOVA was performed using SAM module to extract significantly affected probes across all three sample groups. False Discovery Rate (FDR) cut-off (10%) was used to generate the lists of differentially expressed (DE) genes.

Single microarray samples (see above, tumor RNA isolation) derived using xenografts from EV, TIMP-2 and Ala+TIMP-2 tumor cell lines were processed and normalized using protocol described above. A fold change analysis was performed between the three sample classes.

DE gene lists from SAM and fold change analyses were used to extract enriched pathways on Ingenuity Pathway Analysis (IPA) software. An unbiased hierarchical clustering on the median centered and normalized DE genes was performed on Cluster version 3.0 and visualized on Java TreeView version 1.16r2.

### A549 Xenografts and tumor processing

The inoculation of A549 EV, TIMP-2 and Ala+TIMP-2 cells into NOD-SCID mice (n=15 per group) and growth curves are described elsewhere [12, 21]. The mice were sacrificed 21 days post injection, and tumors were harvested. Necrotic tissue was removed, and the tumors were either processed immediately or stored in RNAlater (Ambion, Grand Island, NY) for RNA extraction.

## Supplementary Figures


