# Supplementary material for: TIMP-2 modulates cancer cell transcriptional profile and enhances E-cadherin/beta-catenin complex expression in A549 lung cancer cells
Source: Oncotarget. 2013 Jan 27;4(1):163–73. doi: 10.18632/oncotarget.801 (PMC3702216; doi:10.18632/oncotarget.801)
Supplement: Supplementary file 1 [file oncotarget-04-163-s001.pdf]

TIMP-2 modulates cancer cell transcriptional profile and enhances E-cadherin/beta-catenin complex expression in A549 lung cancer cells - Bourboulia et al

A.

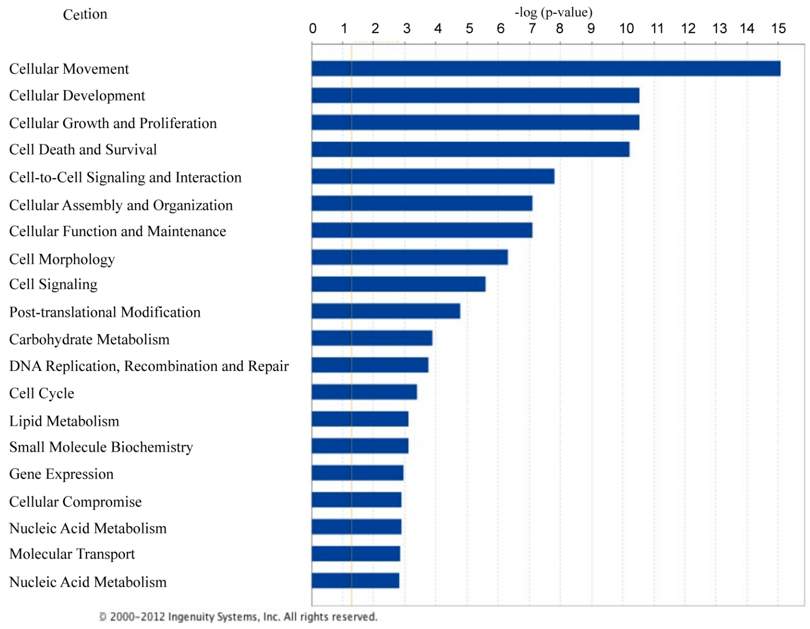

B.

ONTOLOGY CATEGORY	FUNCTIONS ANNOTATION	TIMP-2 vs EV	
		p-value	Predicted Activation State
Tissue Development	development of organ	7.04E-06	Increased
Cellular Movement	cell movement of prostate cancer cell lines	1.27E-05	Increased
Cellular Growth and Proliferation	proliferation of connective tissue cells	2.76E-04	Increased
Renal and Urological System Development and Function	cell movement of kidney cell lines	8.32E-03	Increased
Connective Tissue Development and Function	focus formation of fibroblast cell lines	6.01E-03	Increased
Cellular Development	epithelial-mesenchymal transition	5.95E-03	Increased
Neurological Disease	experimental autoimmune myasthenia gravis	4.55E-03	Increased
Tissue Morphology	strength of tissue	9.76E-03	Decreased
Lipid Metabolism	synthesis of lipid	5.72E-03	Decreased
Cancer	metastasis of tumor cells	4.02E-03	Decreased
Cardiovascular Disease	heart disease	1.70E-03	Decreased
Carbohydrate Metabolism	phosphorylation of carbohydrate	1.22E-02	Decreased
Cell Death	cell death of muscle cells	1.01E-02	Decreased
		Ala+TIMP-2 vs EV	
		p-value	Predicted Activation State
Cell-To-Cell Signaling and Interaction	binding of connective tissue cells	2.09E-04	Increased
Neurological Disease	hyperalgesia	1.87E-02	Increased
Cancer	carcinoma	2.59E-07	Decreased
Cancer	solid tumor	1.55E-07	Decreased
Tissue Development	differentiation of osteoclasts	1.40E-05	Decreased
Cell Morphology	volume of cells	2.13E-04	Decreased
Nervous System Development and Function	development of nerves	3.02E-04	Decreased
Cell Death	survival of epithelial cells	1.93E-02	Decreased

Supplementary Figure 1: (A) List of prominent biological and cellular functions over-represented across TIMP-2 and Ala+TIMP-2 in A549 cells. A threshold value of $p<0.05$ is shown on the bar chart. (B) Functions predominantly affected by TIMP-2 and Ala+TIMP-2 overexpression and the predicted effect are shown.
